# Implementing Standardised Delirium Screening Using the Four A's Test (4AT) in the Emergency Department: A Quality Improvement Project

**DOI:** 10.7759/cureus.83361

**Published:** 2025-05-02

**Authors:** Danielle C Durant, Payal Karia

**Affiliations:** 1 Accident and Emergency, Addenbrooke's Hospital, Cambridge University Hospitals National Health Service (NHS) Foundation Trust, Cambridge, GBR; 2 Geriatrics, Colchester University Hospital, Essex, GBR

**Keywords:** 4at, acute delirium, adult emergency department, delirium screening, locum, quality improvement projects, rotational training, standardisation

## Abstract

Introduction: Elderly patients are at risk of developing an acute, temporary change in cognition referred to as delirium. Those identified as at risk should be screened promptly when presenting to the emergency department to ensure early recognition and reversal of the underlying cause to improve outcomes. Our quality improvement project (QIP) aims to introduce standardised delirium screening into the emergency department with a target of 80% adherence.

Materials and methods: We conducted a QIP at Southend University Hospital emergency department in England from June 15, 2023 until November 7, 2023. We planned to implement the four A’s test (4AT) as the recognised standardised scoring system for delirium screening in those 65 years of age or older. The interventions included a preparatory survey, formal teaching and poster prompts. We performed three cycles of data collection with 50 patients in each set of data. Our target was 80% use of the 4AT.

Results: This QIP demonstrates a modest improvement in the use of a 4AT as the standardised delirium screening in those 65 years of age or older; however, work is still required. The first cycle of 50 patients revealed that 0 (0%) patients had documented use of 4AT for screening. Our second cycle of 50 patients performed after formal teaching to emergency doctors revealed that six (12%) patients had a documented 4AT. The third cycle performed after poster prompts had four (8%) patients having documented 4AT screening. Whilst there is improvement from baseline levels, the initial uptake in the use of 4AT was not sustained.

Conclusion: An improvement in 4AT usage was demonstrated compared to baseline with the most effective intervention being formal teaching; however, the target of 80% was not met. There are notable barriers to the implementation including rotational training and challenging working conditions. This highlights the need for robust quality improvement and systemic level change with consideration given to to implementation of 4AT during nurse triage, perhaps utilising formal teaching.

## Introduction

Managing co-morbid geriatric patients with varied cognitive baselines is becoming increasingly common within an emergency department (ED) setting. Clinicians are assessing for cognitive impairment and this requires standardisation to be performed and recorded reliably. Our quality improvement project (QIP) aimed to standardise delirium screening with a target 80% documentation rate of the four A’s test (4AT) for all patients aged 65 years and older by November 2023. A rate of 80% was selected as this was considered achievable.

There are multiple tools available to us as clinicians to objectively assess a patient for an acute reversible change in cognition, referred to as delirium. These tools can assist in early recognition of a delirious state, as well as allow geriatricians to compare their objective scores during their inpatient stay by monitoring for improvement.

Early identification and monitoring of delirium is important as it prompts clinicians to search for the underlying cause and attempt reversal. Causes commonly include chest infections, urine infections, constipation, hyponatraemia and unfamiliar places. Reversal of these causes leads to resolution of the delirium over the following days to weeks [1].

The National Institute of Clinical Excellence (NICE) guidelines state being aged 65 years and older is a risk factor for delirium [2]. They recommend every patient 65 years of age and older should have a formal delirium screen when first presenting to the hospital. We know early identification leads to improved patient outcomes and reduced length of hospital stay [3]. Further to this, early screening also reduces the quantity of visits to the ED [4]. The responsibility of early recognition falls firmly on emergency clinicians as they are often the first to consult with the patient.

The 4AT is used to screen for delirium and has four domains; alertness, abbreviated mental test four (AMT4), attention and fluctuating course [5]. The 4AT is quick to perform, taking approximately two minutes [6]. It has a sensitivity of 88% for picking up delirium and a specificity of 87-88% [7,8].

At Southend University Hospital, there was no standardised screening test approach to assessing cognition in the ED. Some clinicians would screen with the 4AT, or a different test, or not screen at all. Lack of standardisation has been raised as an issue in previous studies [9,10], making it difficult to track cognitive improvement or decline. The preferred test of our local geriatric department was the 4AT, therefore to improve standardisation and comparison, we selected this screening tool.

## Materials and methods

This QIP was performed at Southend University Hospital, England, a district general teaching hospital.

Preparation: survey (June 15, 2023 to June 30, 2023)

To prepare for improving the screening process, we conducted a survey inviting doctors within our department to share their views on delirium screening. All Southend University Hospital resident emergency doctors were invited via email to participate anonymously. We received six responses. Six (100%) of those who responded could list the four categories of the 4AT, showing it is well-recognised and memorable. Three (50%) participants stated they perform the 4AT when admitting patients over the age of 65. When asked how to improve our usage, the top-rated answers were teaching and the use of posters. Two (33%) respondents believed doctors should be documenting the 4AT for patients of 65 years of age, while four (66%) stated they believed nursing staff would be best placed to do this at triage.

Baseline data collection: cycle 1 (July 6, 2023)

For our first cycle of data collection performed on July 6, 2023, we randomly selected a 48-hour period and chronologically selected the most recent 50 patient notes in that period of ED attendees. Inclusion criteria for all data collection was 65 years of age or older. The exclusion criteria for all data collection was triage-only assessment as we used the doctor's notes for data collection. All data collection was unblinded. We checked the ED doctor's records for proof of a 4AT score being recorded.

Intervention 1: teaching session (July 26, 2023)

Taking into account the feedback we had received on our survey, our first intervention was a teaching presentation given at our morning handover meeting to emergency doctors. This session was attended by resident emergency doctors of varying grades and consultants. We did not take attendance numbers. To account for those not present we circulated this presentation via email to all members of the ED doctors team to ensure everyone had equal access to teaching.

Our presentation discussed the four core components of the 4AT and the NICE guidance. We provided in-house training and answered any queries regarding the usage of the 4AT. We suggested further standardisation of recording the 4AT in the “Neurology” section of the clerking proforma.

The evidence of the content of the teaching slides can be seen in Appendix A-F.

Data collection: cycle 2 (September 26, 2023)

The second round of data collection took place on September 26, 2023. We randomly selected a 48-hour time period and chronologically selected the most recent 50 patient notes of ED attendees within that period. We checked their emergency clerking notes for evidence of a recorded 4AT and assessed for improvement.

Intervention 2: posters (October 12, 2023)

**Figure 1:**
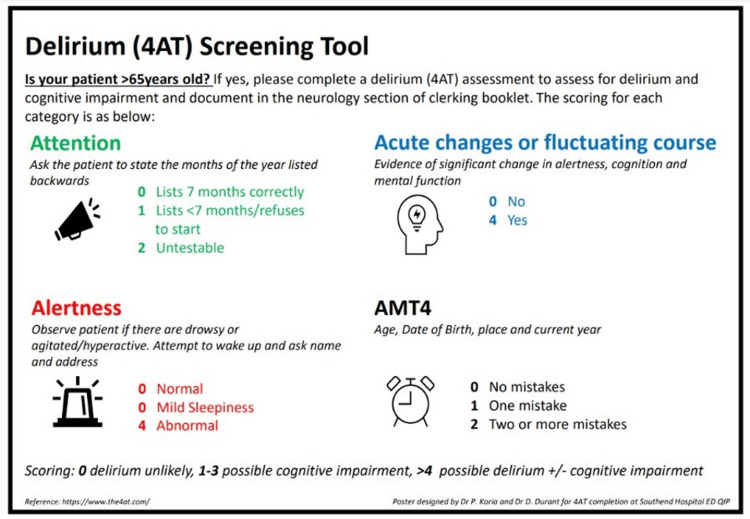
Poster used for prompting 4AT usage detailing components of the 4AT 4AT: four A's test, AMT4: abbreviated mental test four Image credits: Payal Karia, Danielle C. Durant Based on: https://www.the4at.com [5]

For our second intervention, we took on board the other survey suggestion and placed posters throughout the ED detailing the 4AT as a tool and prompt. Five posters were placed. Areas included the doctor's office above computers at eye level, the handover room for morning meetings and walls in majors and minors areas. Copies of the poster (Figure 1) were circulated to all ED resident doctors via email.

Our goal was that after teaching, we would provide a prompt and a tool to calculate the risk of delirium by using the poster as a guide.

Data collection: cycle 3 (November 7, 2023)

For the last round of data collection we randomly selected a 48-hour time period and chronologically selected the most recent 50 patient notes of ED attendees in that period. We reviewed the notes to evaluate for improvements and reflected on issues that had arisen from this cycle.

Data analysis

All data collected from patient ED notes were checked by two separate clinicians to ensure accuracy of patient age and use of 4AT. This analysis aimed to assess completeness and adherence to the new local standard and identify areas for further improvement.

Ethical approval

This QIP was registered with the Audit & QIP Department at Southend Hospital and underwent local ethical approval as part of the QIP process. Formal ethical approval was not sought as this QIP did not meet the local requirements to do so as it was for quality improvement. Patient identifiers (patient name, NHS number or initials) were not used in recordings from patient notes, 4AT outcomes were recorded alongside numbers 1-50.

Evaluation

Evaluation of collected data focused on assessing the impact of the interventions. This included reviewing if the 4AT was documented as used, reviewing feedback, identifying strengths and weaknesses of intervention choice, and gathering suggestions for future improvement of the screening tool.

## Results

Table 1 demonstrates the summarised results of our QIP.

**Table 1:** Progress of 4AT documentation in emergency department notes across three cycles 4AT: four A's test

Cycle	N (%) of patients with a 4AT documented in emergency department notes
First cycle	0 (0%)
Second cycle	6 (12%)
Third cycle	4 (8%)

In our first cycle, of the 50 patient notes checked 0 (0%) had a documented 4AT score. This was prior to any intervention and demonstrated that in clinical practice, NICE guidance was not being followed to screen for delirium in those 65 years of age and older.

After our teaching intervention, in our second cycle, this improved to six (12%) out of 50 patient notes having a documented 4AT score. This was an improvement of six (12%), showing that education around the topic does produce improved results.

We completed a further intervention of posters. Following this, our third cycle assessed 50 patient notes and demonstrated that four (8%) had a documented 4AT score. This was a decrease of two (4%) compared to the previous cycle. This reduction is notable and there are important reasons why this occurred, which have value for reflection of all QIPs.

## Discussion

Our results demonstrated an overall improvement in screening with 4AT from zero (0%) to six (12%). This improvement was reduced in the next cycle demonstrating four (8%) patients were screened using the 4AT. We did not reach our 80% rate of screening goal for those 65 years and older. This proves quality improvement is still required in this area.

We know from the literature that ED is not good at screening for delirium. Meged-Book et al. evidenced that less than 1% of patients had a delirium diagnosis during their presentation in ED [11]. This conflicts with our knowledge from other studies that the rate of delirium in ED patients is approximately 17.2% [12], a gross level of underdiagnosis. The primary cited reason from the Meged-Book et al. study for low pick-up was no evidence of a delirium screening tool being used in the patient notes [11].

A similar study performed found an initial 4AT usage of 0% in the emergency department [13]. After one round of intervention, this increased to 16%, then subsequently to 82%, and 92% in the cycles following. The interventions involved education and posters. However, they also involved a department central whiteboard in situ to record the 4AT for each patient, increasing the active participation of clinicians in recording, likely improving outcomes. This is an element that could be adopted in the future for quality improvement in other centres.

Not all quality improvements were as successful in their implementation. The British Geriatric Society Autumn Meeting showcased a QIP detailing a teaching programme to improve the use of 4AT [14]. They collected data in the medical assessment unit which showed the same trend as our results. At baseline, 25% of those 65 years old age and older had a recorded 4AT. With intervention this raised to 41.3%; however, this dropped back to 26.9% when there was no active teaching to reinforce its use. This is comparable to the improvement drop-off we saw in our third-cycle results [15].

From further studies, we know that screening for delirium is worse in the emergency department than in the general wards. In their initial audit cycle, Lafarga-Molina et al. had 0% compliance with delirium screening in ED and general medical wards [16]. In their final cycle, they only achieved a 32% rate of delirium screening in the general wards, with worse numbers recorded in the emergency department due to worse conditions. Of the notes assessed in ED, notably, it was the nursing staff that screened regularly, not the doctors.

There are multiple barriers towards improving the use of 4AT within our department. Four (66%) of our initial survey participants stated they believed nursing staff would be best to perform this screening at triage, therefore some doctors may feel delirium screening is not part of their role. Perhaps future research could consider looking at implementing standardised delirium testing in initial triage nursing paperwork. There is evidence that 4AT screening does not take any more time to perform than conventional nursing triage [17], supporting this idea.

Another important factor that likely harmed the improvement of 4AT usage was the nature of the temporary workforce within the doctor cohort. Rotational doctors swapped during our data collection, therefore many of those we targeted with our teaching session would have moved on to other jobs, and new doctors, including locum doctors, would not have received our teaching. We know that rotational training has been shown to cause a poor sense of stability for the doctors affected [18], but what is lesser discussed is the impact this has on our services and their sustained improvement.

This is a valuable learning point when taking QIPs into account within the healthcare system. The nature of rotational training and temporary workforces within the doctor cohort means that improvements are challenging to initiate and maintain. Often the doctors performing the quality improvement also rotate, so maintenance of an improvement is difficult to achieve.

There are limitations to our QIP. Our survey consisted of six participants, meaning the survey results cannot be generalised to larger cohorts and may have not been fully representative of opinions and 4AT knowledge. Our data collection was performed in a single institution, making it less generalisable to other hospitals and non-teaching hospitals.

Our patient selection method involved a randomly selected time point and then assessing the most recent 50 patients in that timeframe fulfilling our inclusion criteria. This is quasi-randomisation and cannot be considered true randomisation. Timeframe selection results in temporal bias, in that patients seen in one time period may differ systematically from those in other time periods, reducing generalisability. Event-driven variation including staff shortages are not accounted for in this method, this could have affected rates of documentation during busy working conditions.

With regard to our interventions, the use of posters, whilst a common intervention in healthcare, has limitations. They target passive engagement, in busy working environments like the ED people may not actively engage. Environments are often saturated visually with other posters reducing visibility. We controlled these aspects by strategically placing posters at eye level on uncluttered areas of the wall. We were concise with our message and designed our poster so it could be used as a tool to perform the 4AT, to maximise active engagement.

There are also limitations to our teaching intervention. As it was a one-time exposure this may not be enough to induce change. We controlled this by having interactive engagement in the session for active learning. Attendance was not recorded at the session and due to shift patterns, many of our target audience were not directly involved. We improved exposure by emailing all ED doctors the contents of our QIP and slides. Single teaching sessions often cause short-term impact, which is reflected in our results.

We did not perform statistical analysis on our results, this opens up the risk of subjective interpretation and bias. Future study design could be improved with the use of simple statistical analysis. Our result recording consisted of only recording of the final 4AT score, we did not assess the method of calculating to check for reliability. This could be improved by testing colleagues' methods of calculating to ensure accuracy.

For future quality improvement, care should be taken to implement true randomisation methods and more active and repetitive learning processes. The teaching session demonstrated the largest improvement of 4AT documentation, ensuring larger cohorts of doctors or nurses receive this teaching would likely improve uptake and maintenance of results. Targeting nurses would reduce the impact of rotational training on results impact, perhaps with a dedicated section in nurse triage proformas to document 4AT as a prompt.

## Conclusions

This QIP shows education and prompting of emergency clinicians to perform standardised delirium screening leads to improved usage and documentation of the 4AT. Our most successful intervention was teaching with active participation. However, our project and many others suffer from difficulty maintaining improved changes. A key reason for this is the nature of rotational doctor workforces and temporary staffing.

Further improvement in the implementation and maintenance of delirium screening tools is required in the emergency setting. Improving cohort reach of teaching and focusing on introducing screening into nurse-led triage as a standard may improve future outcomes. This would mean rotational training does not impact the maintenance of screening after implementation.

## Figures and Tables

**Figure 2:**
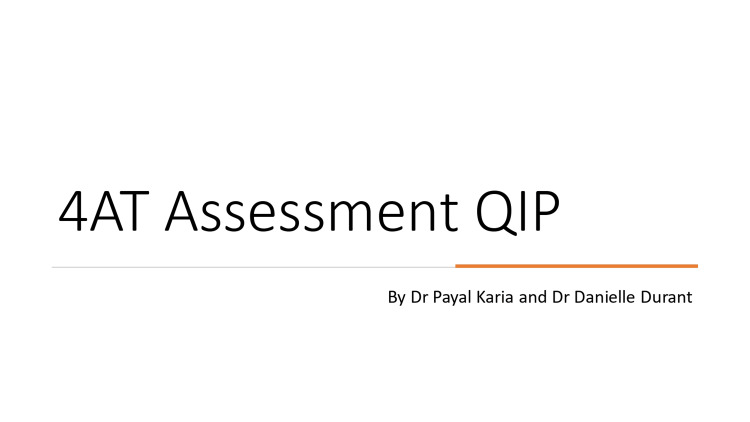
ED teaching presentation slide 1 ED: emergency department, 4AT: four A's test, QIP: quality improvement project

**Figure 3:**
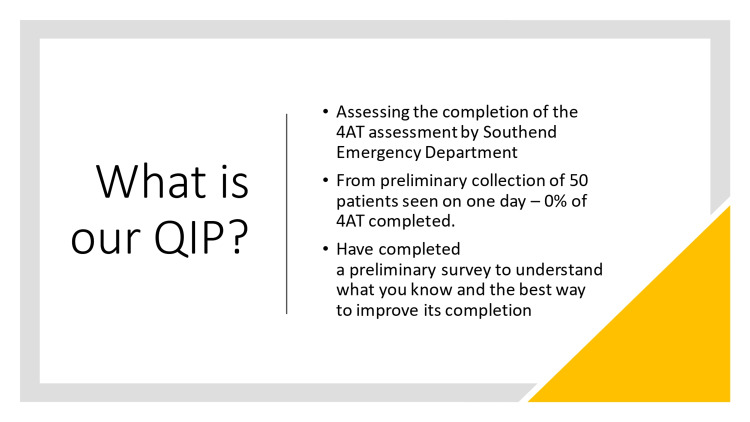
ED teaching presentation slide 2 ED: emergency department, 4AT: four A's test, QIP: quality improvement project

**Figure 4:**
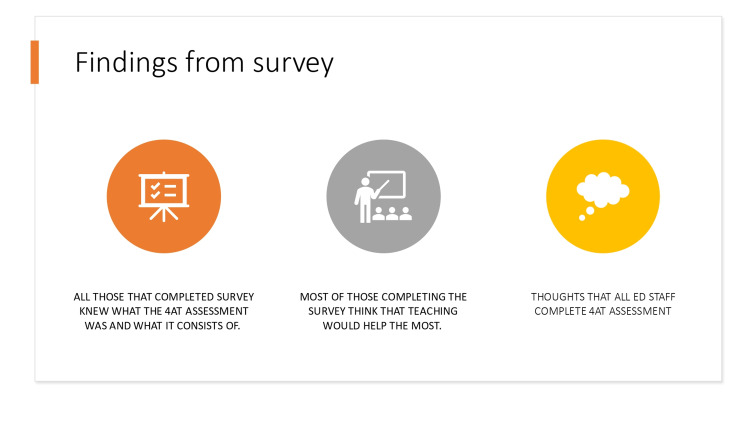
ED teaching presentation slide 3 ED: emergency department, 4AT: four A's test

**Figure 5:**
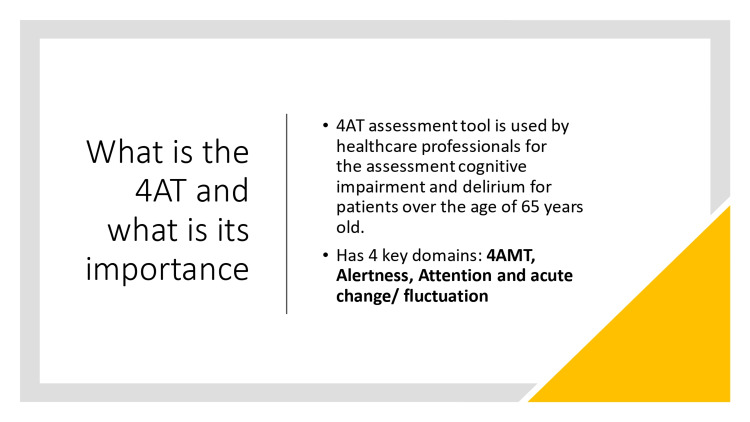
ED teaching presentation slide 4 ED: emergency department, 4AT: four A's test, 4AMT: abbreviated mental test four

**Figure 6:**
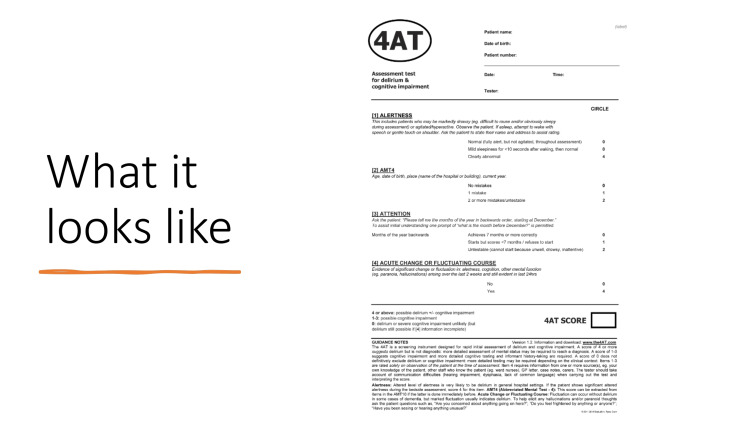
ED teaching presentation slide 5 ED: emergency department, 4AT: four A's test

**Figure 7:**
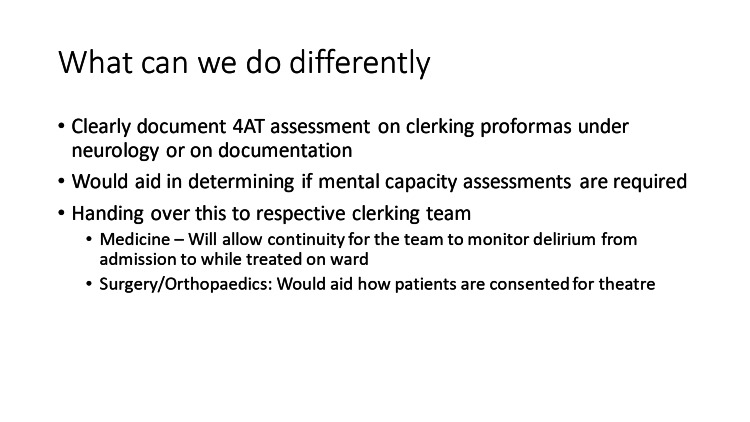
ED teaching presentation slide 6 ED: emergency department, 4AT: four A's test

## Appendices

Appendix A 

Appendix B 

Appendix C 

Appendix D 

Appendix E 

Appendix F 
